# Successful Treatment of a Patient with COVID-19-Induced Severe ARDS, Pneumothorax, and Pneumomediastinum with Awake vv-ECMO Implantation

**DOI:** 10.1155/2022/6559385

**Published:** 2022-08-10

**Authors:** Julian Umlauf, Stefanie Eilenberger, Oliver Spring

**Affiliations:** Department of Anaesthesia and Surgical Intensive Care Medicine, University Hospital Augsburg, Stenglinstr. 2, 86156 Augsburg, Germany

## Abstract

Management of acute respiratory distress syndrome (ARDS) caused by severe acute respiratory syndrome coronavirus 2 (SARS-CoV-2) is still a challenge for the staff on intensive-care units (ICU's) around the world. Many of these patients are treated with invasive ventilation. Sometimes, the occurrence of pneumothorax and/or pneumomediastinum can complicate the course of the disease because initiation of invasive ventilation might be fatal in those patients. Venovenous extracorporal membrane oxygenation (vv-ECMO) is increasingly used to prevent patients with severe ARDS from hypoxia. However, clear recommendations for or against the initiation of vv-ECMO in awake patients are currently lacking. We present the case of a 42-year-old patient with COVID-19-associated severe ARDS, pneumothorax, and pneumomediastinum. To preserve sufficient oxygenation and to avoid invasive ventilation, we implanted a vv-ECMO while the patient was awake. The patient recovered and was discharged home 41 days after transfer to our hospital. We therefore suggest that awake implantation of vv-ECMO might be useful in a subgroup of patients with severe ARDS caused by SARS-CoV-2. However, further evidence is needed to verify our hypothesis.

## 1. Introduction

Coronavirus disease 2019 (COVID-19) caused by severe acute respiratory syndrome coronavirus-2 (SARS-CoV-2) remains challenging for intensivists around the world. The proportion of patients receiving invasive mechanical ventilation (IMV) ranges from 29.1% [1] to 89.9% [2], and a systematic review including 69 studies (*n* = 57420 patients) found that almost half of the patients receiving IMV died [3]. This might also be the case due to ventilator-induced lung injury (VILI) caused by barotrauma, as the incidence of barotrauma appears to be increased in patients with COVID-19 on IMV compared to patients on IMV without COVID-19 [4]. Conclusively, aiming for prevention of lung injury caused by IMV in these patients seems axiomatic.

If the occurrence of pneumomediastinum and/or pneumothorax contributes to high mortality is currently unclear, data are limited to case reports or case series, respectively: a literature review found 35 case reports of spontaneous pneumomediastinum in COVID-19 patients with a mortality rate of 28.5% (10 of 35) [5]. Another retrospective case analysis including 71 patients found that the occurrence of pneumothorax seems not to be an independent risk factor for 28-day mortality [6]. However, in patients with pneumomediastinum and/or pneumothorax, implementation of IMV might be fatal due to the expected worsening of the respective condition.

To prevent patients from hypoxia in severe acute respiratory distress syndrome (ARDS), venovenous extracorporeal membrane oxygenation (vv-ECMO) is increasingly used [7]. A multicenter study including critical ill adults with severe ARDS due to COVID-19 showed a lower in-hospital mortality in patients who received vv-ECMO within seven days of admission to the intensive care unit than patients who did not [8]. However, clinical data describing the application of vv-ECMO in awake patients for prevention of IMV itself is scarce [9].

Hereinafter, we describe the successful treatment of a COVID-19 patient with severe ARDS, spontaneous pneumomediastinum, and pneumothorax using awake implantation of vv-ECMO.

## 2. The Case

**Figure 1:**
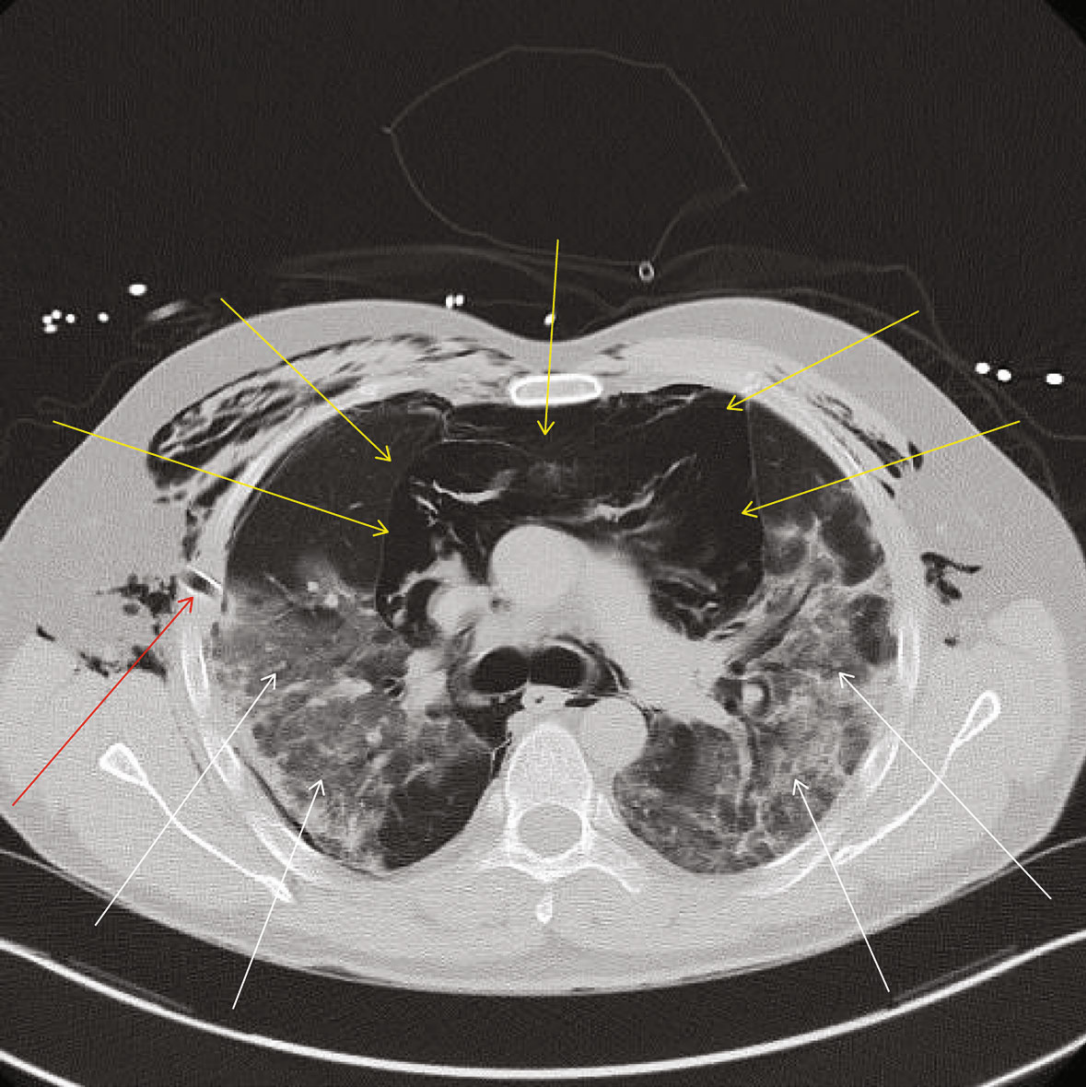
Initial chest CT-scan at the level of tracheal bifurcation directly after transfer to our ICU. The white arrows indicate bilateral ground glass opacities. Yellow arrows display the pneumomediastinum. The red arrow points at the thoracic drainage which was placed during the stay in the referring hospital.

A 42-year-old patient (body mass index 23 kg/m^2^) was transferred from another hospital to our intensive care unit (ICU). He was first tested positive for COVID-19 eight days before he was admitted to our unit. Besides a fructose intolerance, his medical history was empty. At admission to the first hospital, he presented himself with dyspnea and intermittent episodes of fever (temperature maximum 38.2°C). The patient was treated with dexamethasone 6 mg i.v., and intermittent noninvasive ventilation (NIV) was started due to ARDS and dyspnea. During the stay, the patient developed a spontaneous right side pneumothorax and a massive pneumomediastinum. A thoracic drainage had been placed in the right hemithorax, and the patient was transferred to our unit seven days after admission. A chest CT after transfer indicated bilateral ground glass opacities with consolidations and pneumomediastinum (Figure 1).

**Figure 2:**
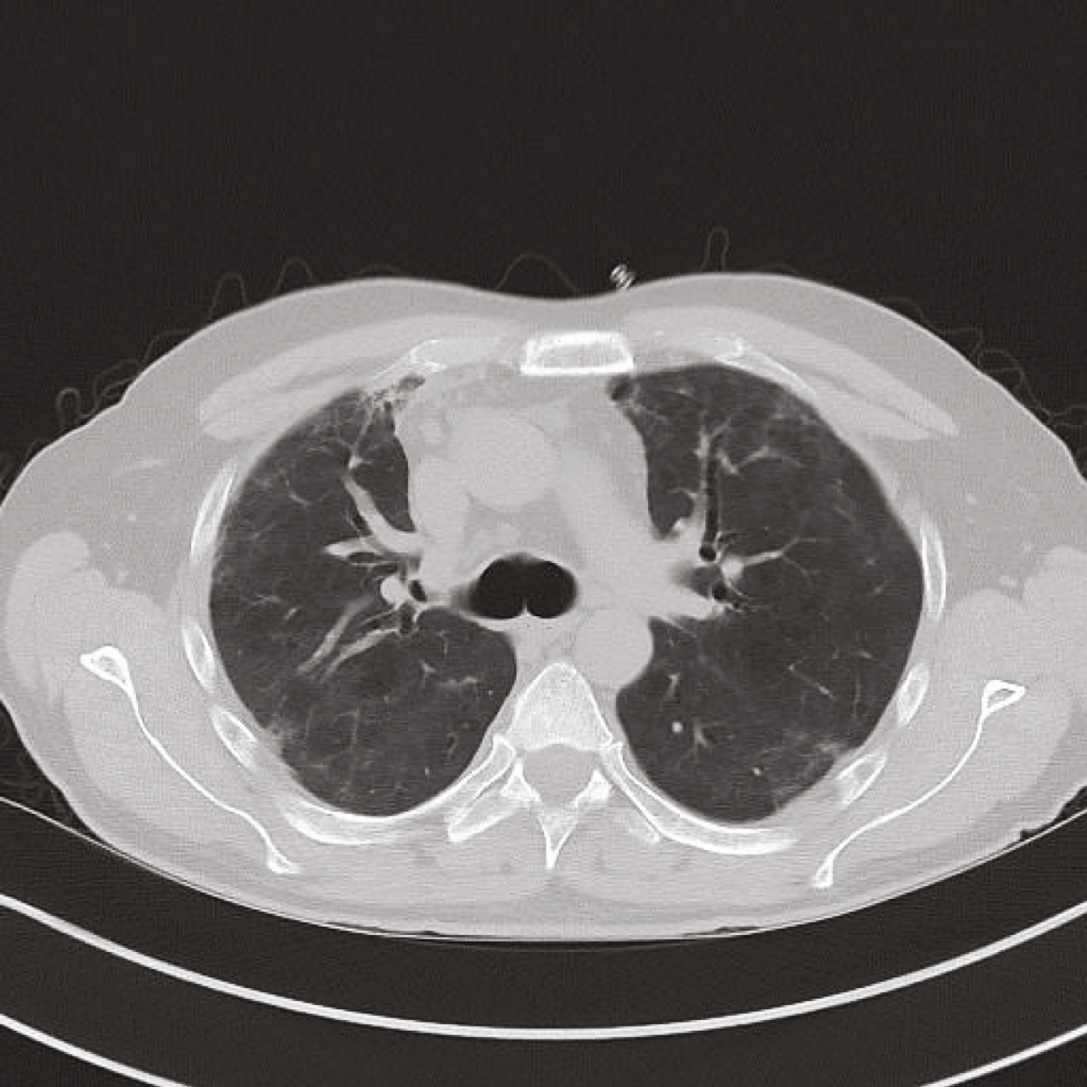
Chest CT-scan 31 days after ECMO implantation, the same level as Figure 1. The pneumothorax and pneumomediastinum completely abated, and the ground glass opacities profoundly receded.

To avoid worsening of the pneumothorax and pneumomediastinum, respectively, NIV-therapy was terminated. The patient received high flow oxygen therapy, and intermittent prone positioning was initiated. PaO_2_ levels under 90% FiO_2_ and a flow rate of 45 l/min ranged from 70 to 80 mmHg, and PaCO_2_ levels were at 35-38 mmHg. Laboratory parameters at admission to our unit were the following: leukocytes 17.22/nl, CRP 4.93 mg/dl, PCT 0.042 ng/ml, LDH 704 U/l, and D-dimers 26049 ng/ml. Intravenous application of dexamethasone was continued. In addition, the patient was treated with budesonide p.inh. and loop diuretics i.v. to achieve negative fluid balance. 48 hours after admission to our ICU, FiO_2_ was reduced to 65% and the flow to 35 l/min. PaO_2_ level was at 63 mmHg, and PaCO_2_ was at 34 mmHg. Five days after transfer, we increased oxygen supplementation due to worsening hypoxia. Eight days after transfer, FiO_2_ was at 100%. The respiratory rate increased to 35/min, and ventilation mechanics worsened. Prone position therapy that had initially enhanced PaO_2_ levels and oxygen saturation, respectively, did not improve oxygenation anymore. PaO_2_ level was at 60 mmHg and PaCO_2_ at 35 mmHg. Due to the massive pneumomediastinum, we refrained from orotracheal intubation and implanted a vv-ECMO in the patient, who was conscious at that time. The cannulas were placed in the right femoral vein and the right internal jugular vein, respectively. During the implantation procedure, we used remifentanil for analgosedation. A short episode of tachyarrhythmia during implantation was treated with 5 mg of metoprolol i.v. Directly after the implantation, PaO_2_ level was at 54 mmHg. ECMO support started with 3.5-3.6 lpm, FiO_2_ 100%, and a purge gas flow of 4 l/min. The high flow oxygen therapy continued with FiO_2_ at 100%. Although a chest X-ray 48 hours after ECMO implantation showed a notable increase of lung infiltrations, PaO_2_ levels increased to 83 mmHg. On day four after ECMO implantation, inhalative FiO_2_ was reduced to 40%. Seven days after implantation, we started ECMO weaning and reduced the ECMO support to 2.8-3 lpm. PaO_2_ levels remained stable. 17 days after the implantation, ECMO weaning was concluded, and the ECMO-cannulas were removed at the bedside. After explantation, the patient received 5 l O_2_/min via nasal cannula. PaO_2_ level was at 116 mmHg. On day 18 after ECMO implantation, we transferred the patient to a normal ward. A chest CT 31 days after ECMO implantation showed declining ground glass opacities. The pneumothorax as well as the mediastinal emphysema and subcutaneous emphysema was gone (Figure 2).

The patient was discharged home 41 days after transfer to our clinic.

## 3. Discussion

To our knowledge, this is the first published case that describes successful treatment of a patient with COVID-19-associated mediastinal emphysema using vv-ECMO.

The mechanism of occurrence of pneumomediastinum or pneumothorax, respectively, in COVID-19 patients is unclear: on the one hand, barotrauma leading to rupture of alveoles due to positive pressure ventilation might be responsible for mediastinal emphysema: a systematic review including 13 studies (*n* = 1814 patients) found that barotrauma might occur more frequently in patients with ARDS due to COVID-19 than in patients with ARDS from other origins [4]. However, this trial only included patients on IMV; in our case, the patient only received NIV for a relative short period of time. Consequently, despite the fact that barotrauma might have contributed to the occurrence of pneumomediastinum, it appears rather unlikely to be the only reason in the present case.

On the other hand, lung frailty due to COVID-19 itself may also be a contributing factor: a retrospective study including 323 ARDS-patients receiving IMV concluded that pneumomediastinum was sevenfold increased in ARDS due to COVID-19 compared to ARDS from other origins; interestingly, no difference in PEEP, plateau pressure, peak pressure, compliance, and tidal volume/ideal body weight ratio between COVID-19 patients with and without pneumomediastinum was observed [10]. The authors concluded that barotrauma due to IMV might be rather less responsible for the occurrence of pneumomediastinum than lung frailty due to COVID-19. This hypothesis is in line with several case reports of spontaneous pneumomediastinum due to COVID-19 without presence of IMV [11] including the present case.

Evidence of application of vv-ECMO in awake patients is currently shaky [9]. A retrospective case series describing eight cases where awake vv-ECMO was applied in COVID-19 patients with severe ARDS found a mortality rate of only 14.3% (1 patient) [12]. Other single case reports also showed promising results [13–15]. However, a prospective observational trial on four German ICUs found that out of 18 patients that were treated with awake vv-ECMO implantation, 14 were intubated during the further stay on ICU [16].

Besides vv-ECMO, another technique for extracorporal gas exchange has emerged in the past several years: the pumpless interventional lung assist (iLA). While the vv-ECMO drains venous blood through a gas exchange membrane before returning it to the venous system using a pump, the iLA-system works with a gas exchange membrane being integrated within an extracorporal arteriovenous bypass using the arteriovenous pressure gradient [17]. As none of these two systems has proven to be superior over the other one so far and iLA is not available in our hospital, we used vv-ECMO in this patient.

All in all, we hypothesize that the use of vv-ECMO to avoid IMV in patients with pneumothorax and/or pneumomediastinum might be considerable and applicable in a subgroup of patients (e. g., young age, satisfactory psychological compliance, and expected worsening during IMV) with severe ARDS due to COVID-19. Further evidence is needed to confirm or discard our hypothesis.

## Data Availability

No data were used to support this study.
